# Supplementary material for: Content-rich biological network constructed by mining PubMed abstracts
Source: BMC Bioinformatics. 2004 Oct 8;5:147. doi: 10.1186/1471-2105-5-147 (PMC528731; doi:10.1186/1471-2105-5-147)
Supplement: Additional File 2 — The original results of the above study (non-essential files are deleted to keep the file size under the limit set by BMC bioinformatics). [file 1471-2105-5-147-S2.bz2 › chilibotAdditionalFile2/dip10/711ID8679677E18048/html/PABA_PABB.html]

 


 **PABA** and **PABB** 
  
Found 23 abstracts in PubMed, retrieved 10.  
 

 What does Google say? 
 PDF only 
| .edu only 

---

**Interactive relationship** (e.g. stimulation, inhibition, etc)

**Neutral relationship**- We conclude that both  **PabA**  and  **PabB**  have cysteine residues which are essential for catalytic function and or for subunit interaction.  Ref: 7592344 J Bacteriol, 1995
- A gene  **pabB**  encoding the aminase activity of p aminobenzoate  **PABA**  synthase in Lactococcus lactis subsp.  Ref: 8409921 J Gen Microbiol, 1993
- The cloned heterologous  **pabB**  DNA was expressed in amounts that allowed accumulation of excreted  **PABA**  in cultures of S. lividans transformants.  Ref: 8409921 J Gen Microbiol, 1993
- Aminodeoxychorismate ADC synthase  [ **PABB** ]   [ **PABA** ]  converts chorismate and glutamine to ADC and glutamate and is composed of two subunits,  **PabA**  and  **PabB** .  Ref: 7592344 J Bacteriol, 1995
- 4 Amino 4 deoxychorismate ADC synthase  [ **PABB** ]   [ **PABA** ]  converts chorismate and glutamine to ADC and glutamate, and is composed of two subunits,  **PabA**  and  **PabB** .  Ref: 8679677 Biochim Biophys Acta, 1996
- We report that  **PabA**  requires a preincubation with dithiothreitol for maximal activity as measured by its ability to support the glutamine dependent amination of chorismate by  **PabB** .  Ref: 7592344 J Bacteriol, 1995
- A comparison of the structures of these two classes of chorismate utilizing enzymes provides a rationale for the differences in quaternary structures seen for these enzymes, and indicates that the weak or transient association of  **PabB**  with  **PabA**  during catalysis stems at least partly from a limited interface for protein interactions.  Ref: 11841211 Biochemistry, 2002
- In the first step, a glutamine amidotransferase encoded by the  **pabA**  gene generates ammonia as a substrate that, along with chorismate, is used in the second step, catalyzed by aminodeoxychorismate synthase, the product of the  **pabB**  gene.  Ref: 11841211 Biochemistry, 2002
- **PabB**  glutamine enhances the protective effect of  **PabA** .  Ref: 7592344 J Bacteriol, 1995

**Non-interactive relationship** (e.g. studied together, co-existance, homology, etc.)

- The protein encoded by ORF pab shows a clear relationship at the N terminus with  **PabA**  and at the C terminus with  **PabB**  from Escherichia coli, Serratia and Klebsiella.  Ref: 8472954 Gene, 1993
- The increased resistance was attributed to expression of the pair of discrete translationally coupled p aminobenzoic acid biosynthesis genes designated  **pabB**   **pabA**  cloned in the 3.8 kb fragment.  Ref: 11495989 Microbiology, 2001
- We report here the first successful copurification of  **PabA**  and  **PabB**  by gel filtration chromatography.  Ref: 8679677 Biochim Biophys Acta, 1996
- While there is much interest in the mechanism of chorismate aminations, there has been little work done on the ADC synthase  [ **PABB** ]   [ **PABA** ]  reaction.  Ref: 7592344 J Bacteriol, 1995
- Comparison of the deduced amino acid sequence with database proteins indicated that the N and C terminal regions resembled  **PabA**  and  **PabB** , respectively, of numerous bacteria.  Ref: 8704974 Microbiology, 1996
- The overall biochemical reaction catalyzed by the  **PabB**  and PabC components of  **PABA**  synthase is chorismate aq ammonia aq =4 aminobenzoate aq pyruvate aq H 2 O l.  Ref: 11975992 Biophys Chem, 2002
- In contrast, unfused  **PabB**  group and fused  **PabA**  B proteins form a monophyletic group on a branch separate from the chorismate amination subunits of anthranilate synthases, probably reflecting a need for recognition of different positions in the common substrate chorismate.  Ref: 11981873 J Basic Microbiol, 2002
- While various experiments have suggested that  **PabA**  and  **PabB**  act as a complex, attempts to isolate the intact complex have failed.  Ref: 8679677 Biochim Biophys Acta, 1996
- Introducing vectors containing the 3.8 kb NcoI fragment of S. venezuelae DNA into  **pabA**  and  **pabB**  mutants of Escherichia coli, or into the Streptomyces lividans pab mutant JG10, enhanced sulfanilamide resistance in the host strains.  Ref: 11495989 Microbiology, 2001
- The 3840 bp DNA sequence fragment reported here contains a 2199 bp open reading frame encoding a 733 amino acid protein with similarity to the two components of  **PABA**  synthase described for prokaryotes Escherichia coli  **PabA**  and  **PabB** , suggesting that  **PABA**  synthase is bifunctional in yeast.  Ref: 8346682 Yeast, 1993
- Mutated  **PabB**  enzymes fall into three categories according to their properties deficiency of chorismate amination coupled with failure to associate with  **PabA** , deficiency of chorismate amination coupled with retention of  **PabA**  association, and competency of chorismate amination with failure of  **PabA**  association.  Ref: 8679677 Biochim Biophys Acta, 1996
- No inhibition of ADC synthase  [ **PABB** ]   [ **PABA** ]  activity was observed when p aminobenzoate, sulfanilamide, sulfathiazole, and several compounds requiring folate for their biosynthesis were used.  Ref: 7592344 J Bacteriol, 1995
- Using conditions established for maximal activity of the proteins, we measured the Km values for the glutamine dependent and ammonia dependent aminations of chorismate, catalyzed by  **PabB**  alone and by the ADC synthase  [ **PABB** ]   [ **PABA** ]  complex.  Ref: 7592344 J Bacteriol, 1995
- In most bacteria, these two functions are encoded in separate genes e.g.,  **pabA**  and  **pabB**  of E. coli.  Ref: 11981873 J Basic Microbiol, 2002
- Equilibrium measurements were performed on the first partial ADC synthase  [ **PABB** ]   [ **PABA** ]  reaction at T=298.15 K and at pH=8.78.  Ref: 11975992 Biophys Chem, 2002
- The association of  **PabA**  and  **PabB**  is greatly enhanced by the presence of 5 mM glutamine, and by preincubation at 37 degrees C.  Ref: 8679677 Biochim Biophys Acta, 1996
